# Quantitative Expression and Co-Localization of Wnt Signalling Related Proteins in Feline Squamous Cell Carcinoma

**DOI:** 10.1371/journal.pone.0161103

**Published:** 2016-08-25

**Authors:** Antonio Giuliano, Rebecca Swift, Callum Arthurs, Georgina Marote, Francesca Abramo, Jenny McKay, Calum Thomson, Mariana Beltran, Michael Millar, Simon Priestnall, Jane Dobson, Fernando Costantino-Casas, Terry Petrou, Imelda M. McGonnell, Anthony J. Davies, Malcolm Weetman, Oliver A. Garden, John R. Masters, Christopher Thrasivoulou, Aamir Ahmed

**Affiliations:** 1 Department of Veterinary Medicine, University of Cambridge, Cambridge, United Kingdom; 2 Department of Clinical Sciences and Services, The Royal Veterinary College, London, United Kingdom; 3 Division of Surgery, University College London, London, United Kingdom; 4 Prostate Cancer Research Center at the Centre for Stem Cells and Regenerative Medicine, King’s College London, London, United Kingdom; 5 Department of Veterinary Sciences, University of Pisa, Pisa, Italy; 6 IDEXX Laboratories Ltd., Grange House, West Yorkshire, United Kingdom; 7 Dundee Imaging Facility, College of Life Sciences, University of Dundee, Dundee, United Kingdom; 8 Queen’s Medical Research Institute, University of Edinburgh, Edinburgh, United Kingdom; 9 Department of Pathology and Pathogen Biology, The Royal Veterinary College, London, United Kingdom; 10 Department of Comparative Biomedical Sciences, The Royal Veterinary College, London, United Kingdom; 11 Division of Surgery, University College London, London, United Kingdom; 12 University of London, London, United Kingdom; 13 Independent Vetcare, Bath, United Kingdom; 14 Research Department of Cell and Developmental Biology, The Centre for Cell and Molecular Dynamics, Rockefeller Building, University College London, London, United Kingdom; Okayama University, JAPAN

## Abstract

Feline oral squamous cell carcinoma (FOSCC) is an aggressive neoplasm in cats. Little is known about the possible molecular mechanisms that may be involved in the initiation, maintenance and progression of FOSCC. Wnt signalling is critical in development and disease, including many mammalian cancers. In this study, we have investigated the expression of Wnt signalling related proteins using quantitative immunohistochemical techniques on tissue arrays. We constructed tissue arrays with 58 individual replicate tissue samples. We tested for the expression of four key Wnt/ß-catenin transcription targets, namely Cyclin D1 (CCND1 or CD1), FRA1, c-Myc and MMP7. All antibodies showed cross reactivity in feline tissue except MMP7. Quantitative immunohistochemical analysis of single proteins (expressed as area fraction / amount of tissue for normal vs tumor, mean ± SE) showed that the expression of CD1 (3.9 ± 0.5 vs 12.2 ± 0.9), FRA1 (5.5 ± 0.6 vs 16.8 ± 1.1) and c-Myc (5.4 ± 0.5 vs 12.5 ± 0.9) was increased in FOSCC tissue by 2.3 to 3 fold compared to normal controls (p<0.0001). By using a multilabel, quantitative fluorophore technique we further investigated if the co-localization of these proteins (all transcription factors) with each other and in the nucleus (stained with 4',6-diamidino-2-phenylindole, DAPI) was altered in FOSCC compared to normal tissue. The global intersection coefficients, a measure of the proximity of two fluorophore labeled entities, showed that there was a significant change (p < 0.01) in the co-localization for all permutations (e.g. CD1/FRA1 etc), except for the nuclear localization of CD1. Our results show that putative targets of Wnt signalling transcription are up-regulated in FOSCC with alterations in the co-localization of these proteins and could serve as a useful marker for the disease.

## Introduction

Squamous cell carcinoma is the most common oral neoplasm in cats and people [1]. Feline oral squamous cell carcinoma (FOSCC) is a local aggressive tumor with a low metastatic rate (~15–18%) to local or regional lymph nodes but rarely to the lungs, most cats are likely to be euthanized due to the primary tumor before developing metastasis [2]. The etiopathogenesis of feline SCC is unknown, but some factors have been implicated to increase the risk of development of this type of cancer. For example, cats living in households with smokers are considered more at risk of developing FOSCC compared to non-smoking households [3, 4]. Papilloma virus is considered another putative risk factor as in one study 90% of feline cutaneous SCC carried papillomavirus DNA [5].

Some human squamous cell carcinomas (e.g. head and neck squamous cell carcinoma, (HNSCC) or penile squamous cell carcinoma, (PeCa) are similar in characteristics to that of FOSCC. Interestingly, FOSCC has been proposed as a useful spontaneous tumor model for human SCCs [6]. FOSCC has similar tumor biology, molecular markers (p53, VEGF, EGFR) clinical outcome, treatment, and prognosis to the human counterpart [6].

Wnt signalling is a signal transduction pathway, with intracellular free calcium and the 92kDa transcription factor activator, ß-catenin, as two major intracellular transducers [7, 8]. Wnt signalling is known to be dysregulated in many human cancers [9]; one of the key pieces of evidence of the direct involvement of Wnt signalling in carcinoma is in the human colon cancer where mutations in the APCCD1 and AXIN genes that encode for one of the destruction complex proteins (see [10] for a review) are known. There is also evidence of the dysregulation of Wnt signalling proteins in other carcinomas of the breast, prostate, head and neck and penis with an increased expression of proteins involved in the Wnt signalling network [11–13]. Both HNSCC and PeCa exhibit over-expression of key targets of Wnt/ß-catenin transcription targets [13–15]. Dysregulated Wnt signalling has also been implicated in oral SCC [16].

To investigate the role of WNT signalling in FOSCC, we used a quantitative immunohistochemical technique [17], in combination with high throughput unbiased signal quantification and confocal imaging to compare the expression and co-localization of three Wnt ß-catenin transcription targets which we have previously observed as being upregulated in other squamous cell carcinomas, namely, CD1, c-MYC and FRA1 [13] in tissue arrays of normal buccal feline mucosa and naturally occurring FOSCC samples. We have begun to identify the role of Wnt signalling in FOSCC that could be used, eventually, as diagnostic or prognostic markers as well as targets for therapy.

## Materials and Methods

### Sample size calculation and tissue array construction

A sample size calculation was made, based upon our previous work using tissue arrays and quantitative immunohistochemical techniques [13, 17], to differentiate between over-expression using intensity of gray scale signal (with an α and ß of 0.01 and expected difference of 100 mean gray value with standard deviation 50 and ratio of 1/3 between malignant and control groups to be 25 and 9, respectively).

Formalin fixed, paraffin embedded (FFPE) tissue blocks were collected from 2000 to 2008 for pathological examination and curated in archives at the University of Pisa, Italy; the tissue samples were collected for the benefit of the animal for the purposes of diagnostics and/or treatment. A total of 58 individual tissue sample blocks were procured; of these, 31 were classified by 2 pathologists as containing FOSCC; 27 were classified as controls (termed normal or non-neoplastic); the number of individual samples used in this study is well in excess of the required sample size (see above). These samples were arrayed on paraffin blocks (diameter 1mm) in at least duplicates or quadruplicate (where sufficient tissue was available on the original block). Tissue arrays were sectioned onto glass slides and used for haematoxylin and eosin (H&E) staining and immunochemical labeling. Subsequent to H&E staining, the tissue cores were further assessed by a pathologist to verify malignancy. This resulted in a total of 53 FOSCC (from 31 individual samples) and 94 normal control tissue cores (from 27 individual samples) on 3 tissue array paraffin blocks (S1 Fig).

This study design was blind. The teams involved in tissue collection were blind to tissue array construction; individuals involved in tissue array construction were not involved in the staining of the tissue array and those involved in staining were not subsequently involved in the imaging of the tissue arrays. The samples were only decoded once the staining and imaging and quantitative analysis were complete. Histopathological review was performed on H&E stained tissue arrays (S2 Fig).

### Antibodies, optimization and assessment of cross reactivity in feline tissue

Four antibodies against protein targets of ß-catenin transcription were used: CD1 (sc-718, Santa Cruz); FRA1 (ab117951, Abcam); MMP7 (ab 4044, Abcam); c-Myc (NCL-c-Myc; Leica). All antibodies were checked for their cross reactivity, *in silico*, using Basic Local Alignment Search Tool (BLAST) software for use in the feline species. In a limited number of experiments a ß-catenin antibody (ab22656, Abcam) was also used.

The antibodies used for this study were optimized previously using human FFPE tissue samples according to criteria described elsewhere [13], prior to their use in this study. The four target antibodies were re-coded so their identity (other than species raised) was unknown to the operator and a titration run was carried out at a range of dilutions using the antigen retrieval strategy applied to previous human samples. One of the antibodies (MMP7) did not show any reactivity above background levels on feline tissue and was therefore determined not to cross react. There appears to be a specific labeling pattern for the other three antibodies used. A specific staining pattern was determined to have a range of staining intensities across different cells types and tissue components and was significantly of much higher staining intensity than sections where primary antibody was omitted. Omission of the primary antibody gave minimal background staining indicating the detection system employed was not giving any non-specific staining. Stained sections were evaluated for staining patterns in human squamous cell carcinoma samples in the laboratory. An example of the process of antibody optimization is given in S3 Fig and [13].

### 3,3-diaminobenzidine-horse radish peroxidase (DAB-HRP) staining

Staining was performed using Bond automated system according to manufacturer’s protocols; details of the procedure are given elsewhere [13, 17]. CD1, c-Myc and FRA1 were stained in dilutions of 1:1000, 1:250, 1:100 respectively. Briefly, tissue array slides were dried overnight at 60°C, prior to performing antigen retrieval using a pressure cooker (5 min full pressure and 20 min in Novocastra pH 6 retrieval buffer). All immunostaining protocols were carried out on Vison Biosystems Bond X robot using Define (HRP-polymer) detection kits. H_2_O_2_ block, DAB (3,3-diaminobenzidine) and haematoxylin counterstain were performed as per manufacturer protocols. Images (S4 Fig, 1300 x 1030 pixels) for each tissue core were acquired at 10x magnification at standardized settings using a Nanozoomer (Hamamatsu).

#### Immunofluorescence staining using three antibodies in feline tissue arrays

A similar protocol to that described for DAB staining was used except that CD1 (1:100), c-Myc (1:250) and FRA1 (1:100) antibodies were incubated simultaneously and labeled with fluorescence isothiocyanate (FITC), Cy3 and Cy5, respectively [18], according to manufacturer’s protocol. Other details of the protocol have been described previously [13, 17]. Imaging of the whole tissue array was performed using an Axio Scan Z1 (Carl Zeiss) multifluorophore slide scanner.

#### Image particle analysis using ImageJ software

We employed a quantitative, unbiased, automated particle analysis method, used for the analysis of the DAB signal for other tissue arrays [13, 17, 19] to measure the amount of expression of each antibody in pathologically characterized malignant and normal feline tissue using ImageJ software [20]. Macros were written to execute the following sequence of events for acquired jpeg images: 1. Open image 2. Convert to 16-bit image 3. Set threshold 4. Analyze particle (Size 0.5-Infinity, Circularity 0.00–1.00) 5. Save image 6. Save particle information (count, total area, average size and area fraction) into an excel spreadsheet (rsb.info.nih.gov/ij/docs/pdfs/examples.pdf). Units are default ImageJ setting (pixels). Standardization for the amount of tissue per core was also quantified by using the inverse function (Edit>Invert image) in ImageJ with the protocol described above. Quantified DAB signal is expressed as signal / amount of tissue in each tissue core [17]. A contiguous spreadsheet for all tissue cores for normal vs malignant comparison, for different antibodies was constructed. Tests for normal distribution (Kolmogorov-Smirnov test) of the acquired data and subsequent statistical tests for significance of difference between protein expression in malignant and normal cores was conducted using Mann Whitney U test. Mountain plots were constructed and analyzed for area under the curve (AUC) using Origin (Microcal). Receiver Operating Characteristics (ROC) curves were constructed after converting the data to normal distribution using probit using MedCalc software; likelihood ratios were also calculated from the ROC curves using MedCalc.

### Quantitative co-localization of CD1, c-Myc and FRA1

For quantitative co-localization of CD1, c-Myc and FRA1, stained tissue cores (n = 7–18 individual tissue samples) were imaged using an Olympus IX81 confocal system using a dry 40x objective (6x digital zoom). The ‘oif’ files were imported into Huygens Professional software (Scientific Volume Imaging) for image deconvolution for each channel (excitation/emission (nm) FITC = 488 / 519, Cy3 = 559 / 567, Cy5 = 635 / 664). The deconvolved images were used for calculating the Global Intersection Coefficient (GIC), in the Huygens Software analysis tools. GIC is defined as the ratio of intersecting volume to total object volume (giving a proportion of object voxels that contain some intersecting signal). GIC is designed to provide straightforward, intuitive interpretation of two signals intersecting, compared to other measures such as Mander’s or Pearson co-localization coefficients [21, 22].

## Results

### Histopathology

Histopathological assessment was carried out by pathologists involved in tissue procurement and subsequent to tissue array construction. The tissue array slides were stained with H&E and assessed for the presence of tumor in each core (S2 Fig). The diagnosis was incorporated, *post hoc*, into quantitative protein expression analysis. Tumors were confirmed as squamous cell carcinoma (SCC), all SCC samples were moderately to well differentiated. In many core samples, some areas of inflammation fibroplasia and or necrosis were present. Samples with extensive inflammation, necrosis and or fibroplasia were not included in the analysis. (S2 Fig).

### Quantitative analysis of CD1, c-Myc and FRA1 in feline tissue

**Fig 1:**
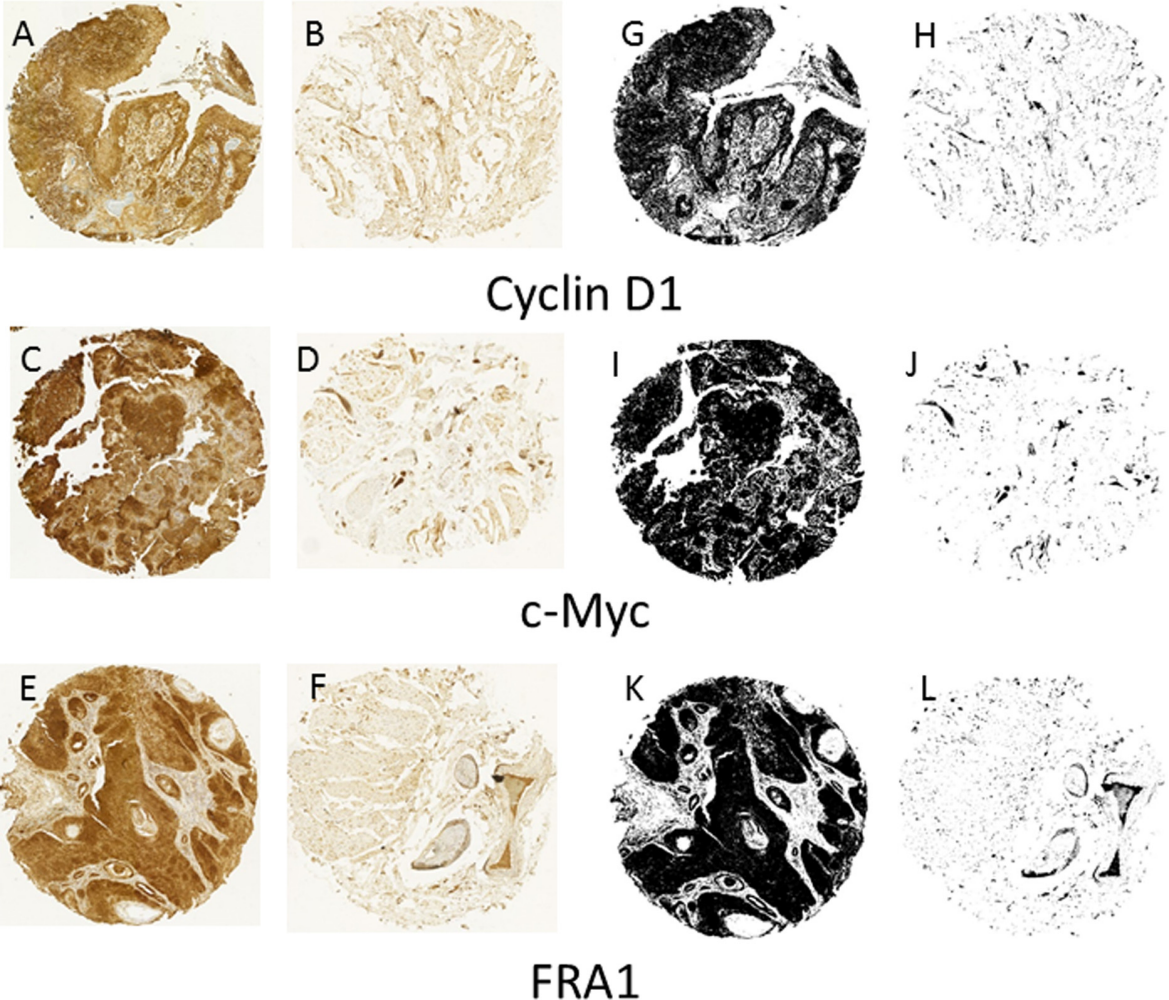
**Staining for CD1, c-Myc and FRA1** in representative feline squamous cell carcinoma (A, C, E) and normal control (B, D, F) samples in a tissue array (58 individual samples were obtained) with DAB-HRP reporter. The tissue array was imaged using a Hamamatsu (Japan) scanner at 10x objective setting and individual core images were used to calculate protein expression (DAB label in RGB format, brown). Colored RGB images were converted to corresponding grayscale images (G-L) for the quantification of the DAB signal using Analyze Particle protocol in ImageJ software (see Materials and Methods –Image particle analysis). Protein expression for CD1, c-Myc and FRA1 was increased in malignant vs normal cores.

**Fig 2:**
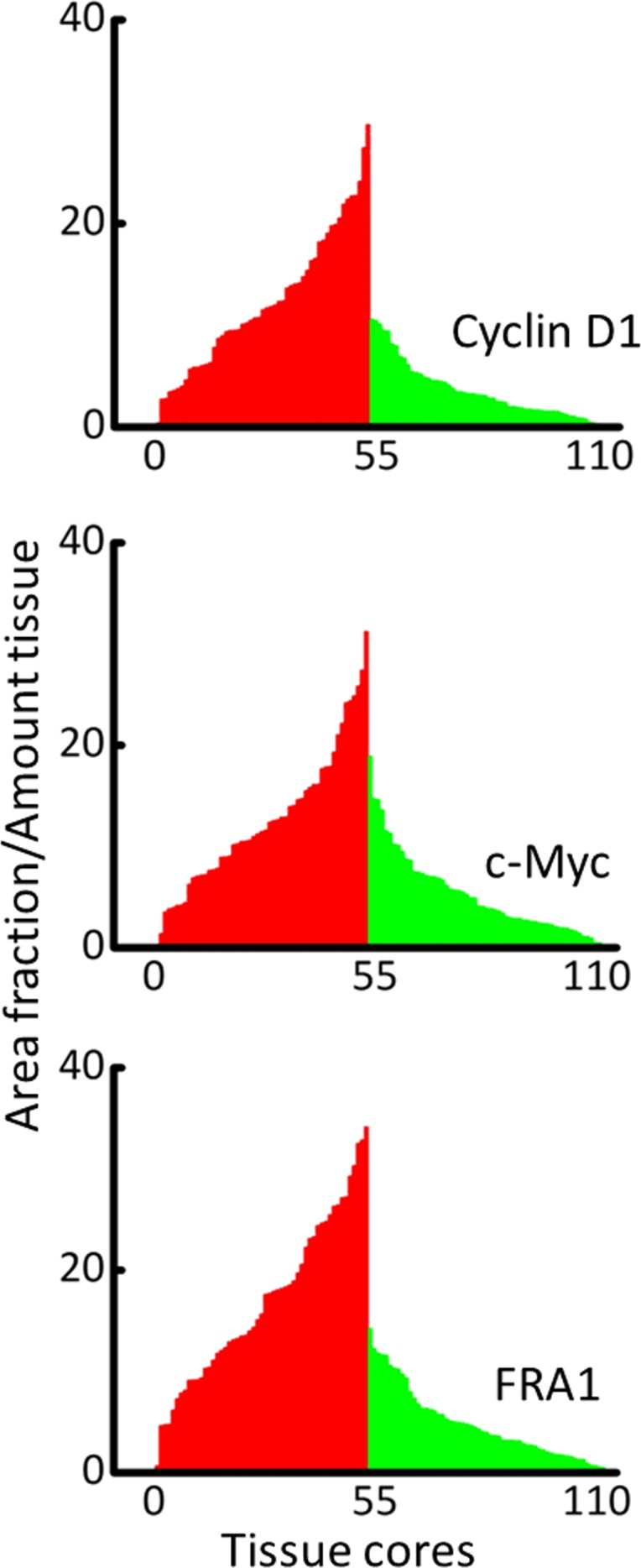
Quantitation of DAB signal in feline tissue array. Total Area (TA) stained (in pixel) with each antibody was quantified using ImageJ software using an unbiased, automated protocol (see Methods); TA is converted to Area Fraction (AF, total area divided by the total pixels in the image). Each bin is data for an individual, malignant (red) or normal tissue (green) core. There is a significant increase in the AUC of protein expression in malignant vs normal feline tissue (p<0.0001).

CD1, c-Myc and FRA1 were expressed in feline tissue cores (>100 tissue cores stained for each antibody); representative images showing the DAB-HRP signal in feline tissue arrays are shown in Fig 1. Representative images were analyzed for each biomarker antibody using the image analysis protocol (see Materials and Methods). The converted grayscale signal (Fig 1) was quantified using a reproducible, semi-automated particle analysis (analyze Particles) protocol; the values for total area (a parameter for antibody expression [17, 19]) are given in Table 1. The data from Table 1 were transformed into plots (Fig 2) and the integration of area under the curve for the DAB-HRP signal (Fig 2) shows an increase (>2 fold) for the expression of CD1, c-Myc and FRA1 in tissue cores that were histopathologically identified as malignant compared to non-malignant (normal) cores (p<0.0001, Mann Whitney U test). These results indicate that the three targets of Wnt-signaling are over-expressed in FOSCC.

**Table 1:** Quantitative analysis of protein expression in FOSCC.

	Total Area / Amount of tissue	Fold increase	Significance (*p<*)
***Control CD1***	62629 ± 53376		
***Malignant CD1***	182012 ± 148535	2.9	0.0001
***Control c-Myc***	93060 ± 77782		
***Malignant c-MYC***	196020 ± 140560	2.1	0.0001
***Control FRA1***	85648 ± 67330		
***Malignant FRA1***	250492 ± 181178	2.9	0.0001

The expression of DAB-HRP labeled proteins (CD1, c-MYC, and FRA1, see Fig 1) was quantified in an unbiased manner by using a reproducible, semi-automated particle analysis protocol (see Methods) using grayscale images of the stained tissue from 147 tissue cores (53 malignant and 94 non-malignant, control tissue cores). Data presented as means ± SD for control vs malignant samples (all grades). Fold increase is relative to control for each protein and significance of difference was calculated using Mann Whitney U test.

**Fig 3:**
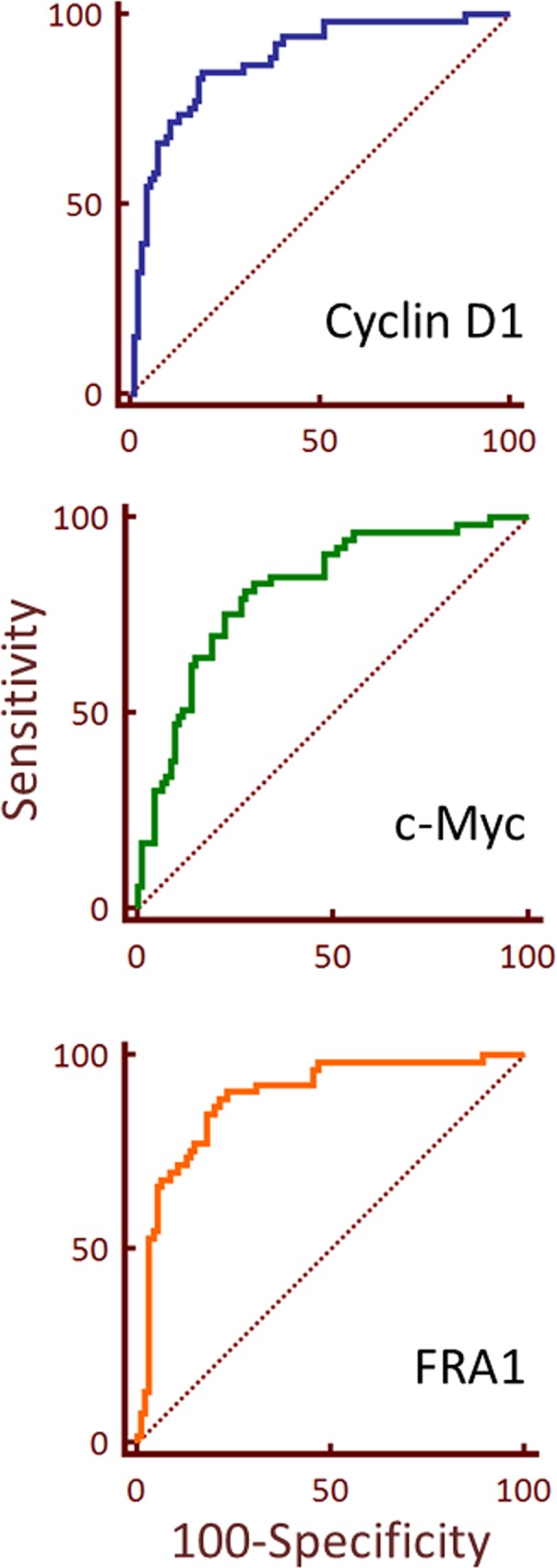
ROC of putative Wnt related proteins in FOSCC. ROC curve demonstrates the discriminating performance of the protein expression in the differentiation between malignant and normal tissue cores using the area fraction (probit) data for (A) CD1, (B) c-Myc and (C) FRA1. The operating characteristic values are given in S1 Table. The dotted line represents the ROC area of 0.5.

The true positive rate (sensitivity) and false positive rate (1-specificity) were calculated from the data used for the ROC curves (Fig 3). The area fraction parameter data was transformed into probit and fitted to a Gaussian function. If the values for area under the curve for an ROC curve fall at or below 0.5, it suggests a marker that cannot distinguish between two categories (e.g. non-malignant and malignant); conversely, values of 1.0 represent high selectivity and sensitivity. The dot plots of the transformed data are given in S5 Fig. We calculated the sensitivity and 1-specificity for area fraction for CD1, c-Myc and FRA1 (S1 Table). Positive likelihood ratios (LR+) for each biomarker at the designated criteria (S1 Table) ranged from 2.9 to 4.5. A likelihood ratio greater than 1 indicates that the result is associated with the presence of disease [23]. CD1, c-Myc and FRA1 also showed LR+ of >10 at various criteria cut off points; LR+s above 10, e.g. for a diagnostic test, are considered to provide strong evidence to rule in disease [23].

### Co-localization of three Wnt signalling proteins, alters between non-neoplastic and SCC feline tissue

We have previously used co-localization of two proteins as an extra measure to distinguish the usability of putative biomarkers of cancer [13, 17]. This is based upon the notion that not only a change in expression but also co-localization of the identified biomarkers may be altered in cancer. We applied this principle to identify if co-localization of CD1, c-Myc and FRA1 were altered in FOSCC.

**Fig 4:**
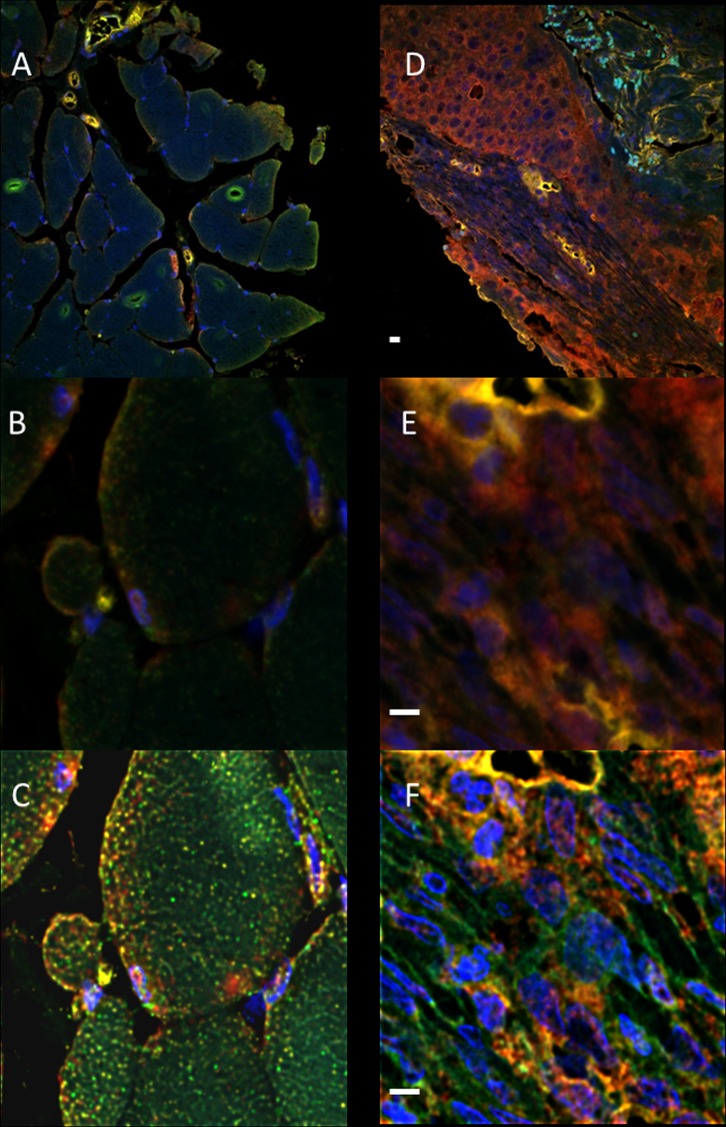
Multi-labeled immunofluorescence for CD1, c-Myc and FRA1. Co-expression of three proteins CD1 (FITC-green), c-Myc (Cy3-red) and FRA1 (Cy5-cyan) in normal (A, B and C) and malignant feline oral tissue cores (D, E and F) and DAPI, counterstain is blue; images are representative tissue cores from the tissue array with over 200 samples. Whole tissue cores (S4 Fig) were imaged using a Zeiss Axioscan Z.1 slide scanner (Carl Zeiss) at 20x magnification with a Calibri.2 LED lights and integration times of the Hamamatsu ORCA Flash4 camera (Hamamatsu Photonics) and fluorescent signals were optimized at the start of study so as to not oversaturate the signal for each antibody. For quantitative co-localization a randomly selected area was imaged using an Olympus IX81 confocal system and a dry 40x objective (A, normal and D, malignant); these areas were further magnified (6x digital zoom) and re-imaged (B, normal and E, malignant). The resulting images were deconvolved using Huygens Software (C, normal and F, malignant) for the calculation of GIC (see Methods). Scale bar = 10μm.

**Fig 5:**
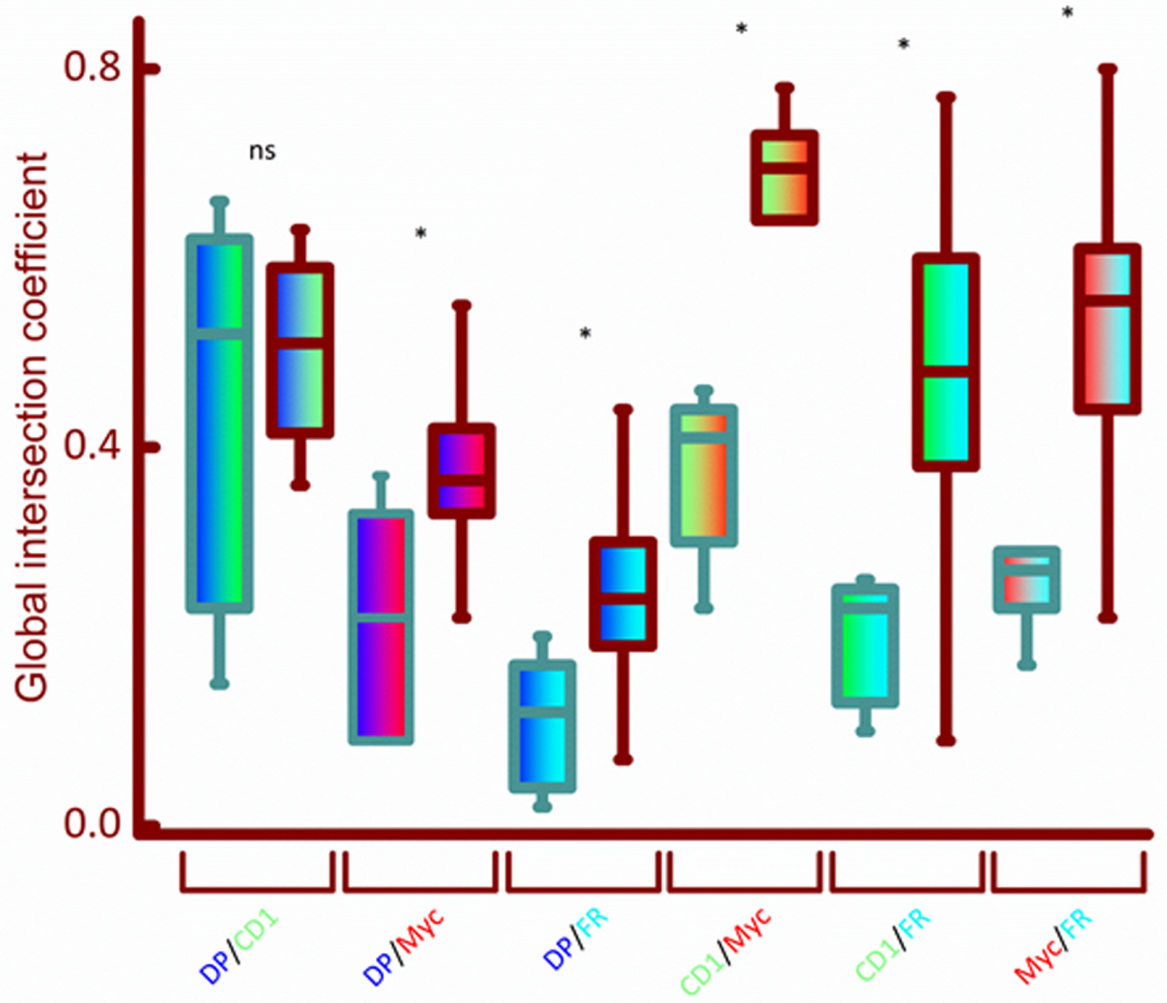
Box plots for differential co-localization of CD1, c-MYC and FRA1 in feline tissue samples. High magnification fluorescent images (e.g. as represented in Fig 4) of feline tissue cores (normal, gray outline; squamous cell carcinoma, brown outlined boxes) were deconvolved and used for the calculation of Global Intersection Coefficient (GIC) using Huygens software. 19 individual tissue samples were used for the three proteins and nuclear stain DAPI (DP) to measure the co-localization of DP/CD1 (blue/green bars), DP/c-MYC (blue/red), DP/FRA1 (blue/cyan), CD1/c-MYC (green/red), CD1/FRA1 (green/cyan) and c-MYC/FRA1 (red/cyan) using GIC (see Materials and Methods). Significance of difference in the GIC for co-localization between normal and FOSCC was calculated using the Mann Whitney U test (* = *p*<0.01, ns = not significant).

We used multi-labeled immunofluorescence using antibodies against CD1, c-Myc and FRA1 (Fig 4 and S4 Fig). The pattern of expression of all three proteins was largely in the epithelium (Fig 4) and similar to that found using DAB-HRP technique (Fig 1). Co-localization, by means of determining the Global Intersection co-localization coefficient, was measured for the protein expression of CD1, c-Myc and FRA1 from fluorescence images (Fig 4) that were de-convolved using Huygens software (see Methods for details). Calculated Global Intersection Coefficient (see Methods) for co-localization for SCC vs non-neoplastic tissue cores are given in Fig 5. There were significant changes in the co-localization of the nuclear stain 4',6-diamidino-2-phenylindole (DAPI) and c-Myc and DAPI and FRA1 fluorophore signals, indicating that the nuclear expression of both of these proteins is increased in malignant compared to non-malignant tissue samples (Fig 5). There was no significant change in the co-localization of the nuclear expression of CD1 in non-malignant and malignant feline samples (Fig 5). The greatest change in co-localization was observed between the signals for CD1 and c-Myc with further significant alterations in the co-localization of CD1 and FRA1 and c-Myc and FRA1 signals (Fig 5). These results indicate that changes in the co-localization of Wnt signalling related proteins may be developed as a useful marker for identifying FOSCC.

## Discussion

Quantitative, molecular measures of the diagnosis and prognosis of cancers could be useful to stratify the type and degree of neoplasia. These measures, through gene expression analysis and medium to large scale immunohistochemical approaches have been used to provide a molecular characterization of human cancers for nearly two decades [24]. Tissue arraying has not been a widely used technique in veterinary medicine, until recently. To our knowledge only a few reports utilize tissue arrays for tumors of the brain [25] breast [26, 27] and for FOSCC, e.g. for the determination of the expression of STAT3 protein expression [28]. No systematic studies have been carried out to quantify the expression of WNT signalling pathways related proteins in FOSCC.

ß-catenin is a transcription factor co-activator protein that is highly conserved during evolution [29]; nuclear translocation of ß-catenin, subsequent to Wnt signalling activation, facilitates gene transcription activation via the TCF/LEF transcription factors [30]. Many of the targets of the ß-catenin/TCF/LEF transcription are proto-oncogenes including c-Myc, c-Jun and FRA1 [30, 31]. It should however be pointed out that these genes may also be targets of transcription via other signaling pathways [32–38]. The over-expression of proteins encoded by Wnt/ß-catenin target genes have been used as a surrogate to determine the role of Wnt signalling in various human carcinomas [12, 13, 17, 39] using tissue arrays. We also conducted experiments to test the nuclear expression of ß-catenin in the FOSCC samples (S6 Fig). The data indicates co-expression of ß-catenin in the nucleus of FOSCC samples.

FRA1 (FOS related activator 1) is a member of the FOS family of transcription factors [40]. FOS proteins were identified, initially, as a viral oncoprotein in avian sarcoma virus 17 [41]. FOS/FRA (and also JUN, ATF and MAF [42, 43]) form a dimeric complex with AP-1 (Activator protein 1) and activate transcription of genes containing AP1 DNA recognition sites [44–46]. Structural and functional alterations of numerous genes, including FRA1, part of AP1 transcription dimer, c-Myc and CD1 are involved in a stepwise transformation of the mucosa to invasive carcinoma in man. The AP1 mediated transcriptional activity regulates numerous cellular process particularly cell proliferation, survival and migration [47, 48]. FRA1 is over-expressed in human cancers (e.g. breast and esophageal squamous cell carcinoma, [49, 50]). Other pathways where FRA1 is implicated include RAS-ERK signalling, which is seen to be upregulated in a variety of carcinoma cell types [34–36]. Our results show for the first time the expression of the Wnt/ß-catenin transcription target FRA1 in FOSCC.

Vmyc (c-Myc) feline and c-Myc human are 93% identical at the protein level (gi163857; gi531813; gi127619 www.ncbi.nlm.nih.gov/protein/127619?report=fasta) NCL-cMyc ab is human c-Myc amino acid 408–420: AEEQKLISEEDLL. The feline v-myc is AgEQKLISEkDLL. (95% identity between human and feline c-Myc—only 25 out of 439 amino acids are different with most being the same class substitution (e.g. acidic for acidic). This high degree of identity is reflected by the signal obtained for this antibody in our experiments (Fig 1). The qualitative expression of c-Myc is increased in 80% of oral squamous cell carcinoma in a South Indian population [51] and it was reported that there is an amplification in the gene copy number in both c-Myc and CD1 in human oral carcinomas [52]. c-Myc is also seen to be upregulated in both multiple myeloma and colon cancer as part of the interleukin signalling pathway [32, 33]. The expression or the role of the c-Myc and CD1 genes has not been investigated in FOSCC around 20% of feline leukaemia virus (FeLV) positive cat T-cell tumors have been shown to involve rearrangements of the Myc gene and around 10% of these possess FeLV recombinant proviruses [53–55]. In contrast, about 20% of naturally occurring feline leukemia virus (FeLV)-positive cat T-cell tumors have been shown to involve rearrangements of the Myc gene, and about half of these possess FeLV-Myc recombinant proviruses.

CD1 is member of a highly conserved cyclin family of genes. Cyclin proteins regulate the activity of cyclin dependent kinases (CDK). CD1 functions as a regulatory subunit of CDK4 or CDK6 necessary for cell cycle G1/S transition. Gene amplification or over-expression of CD1 protein has been demonstrated in many human cancers including head and neck, breast and colon (see [56] for a review). A number of studies have further suggested that CD1 plays a key role in human oral squamous cell carcinoma where over-expression of CD1 is also thought to correlate with detrimental clinico-pathological outcome and poor prognosis [57, 58]. As with the other Wnt/ß-catenin targets discussed above, there is little information regarding CD1 in FOSCC. A recent study, focussed upon STAT3 (a transcription factor complex), investigated the gene expression of CD1 using qPCR in cell lines derived from FOSCC [28] and found that CD1 was expressed in the cell lines tested. This study also used qualitative assessment of protein expression for STAT3 in a tissue array but CD1 expression was not investigated. The expression of CD1 has been investigated in squamous cell carcinoma of the skin using visual analysis, where its expression was found to be rare [59].

There is a need to address diagnosis, prognosis and treatment of companion animal diseases, particularly cancers. Although considerable genetic, environmental and pathophysiological similarities between human carcinomas may exist in companion animal carcinoma, it is essential to develop a better comparative understanding of the companion animal cancers. This will serve the dual purpose of improving companion animal healthcare and may also provide important and useful *in situ* models of cancers in species evolutionarily closer to man than most laboratory models at present. Identifying the role of key signalling pathways in FOSCC, such as the Wnt pathway is, we believe, an important first step. Our results demonstrate that the putative targets of Wnt/ß-catenin transcription are up-regulated in FOSCC as they are in human squamous cell carcinomas [13] and indicate, for the first time, that the Wnt signalling may play a key role in feline carcinoma.

## Supporting Information

S1 Fig Feline tissue arrays were constructed as described in Materials and Methods. Blocks were arrayed with feline tissue samples and also with some human tissue samples for identification. The three slides used in this study (A, B and C) are stained with Haematoxylin & Eosin stain and scanned using a Hamamtsu Nanozoomer scanner are shown here. The original Nanozoomer (ndpi) files were viewed at higher resolution than shown in this figure histopathological analysis using the NDPI.view2 software.
(PDF)

S2 Fig Examples of the histopathological analysis are shown here. Tumor tissue is circled in black and inflammation in red. Present with a multifocal to coalescing pattern is a locally infiltrative, densely cellular neoplastic mass that infiltrates the surrounding stroma. Neoplastic cells are arranged in islands and sheets, within which they exhibit variable degrees of keratinization. Individual neoplastic cells are moderately large, polygonal with moderate to large amounts of cytoplasm. Nuclei are round to oval and contain prominent nucleoli. There is moderate degree of anisocytosis and anisokaryosis. The surrounding stroma exhibits variable amounts of fibrosis and inflammatory cell infiltrate.
(TIF)

S3 Fig An example of the process (e.g. concentration, pH etc) used to optimize the antibodies used in this study in feline tissue. Images were acquired at 10x using a standard bright field microscope. The antibodies were coded (AA xx, A to D) and the experimenters blinded to their identity; negative controls without primary antibodies were used (E). Cross-reactivity of these antibodies was compared by using human tissues in which we have used these, previously (e.g. Arya et al, 2015). Antibody AA33 was not used as the DAB signal for this was similar to the no primary negative control (E).
(TIF)

S4 Fig A feline tissue array showing expression of CD1. (A) The DAB stained slides were scanned using a Hamamtsu Nanozoomer scanner are shown here. The original Nanozoomer (ndpi) files were imaged using the NDPI.view2 software. at a higher resolution (1300 x 1300 pixels) than shown; these higher resolution images were used for quantitative analysis as described in Materials and Methods. (B) Multi-label tissue arrays were stained with three antibodies and counterstained with DAPI, simultaneously, and imaged using an Axioscan (Zeiss). The signal for the four fluorophores is shown in this composite image for one slide.
(PDF)

S5 Fig Dot plots of the distribution of quantitative signal for DAB for each antibody used in this study. Total area (TA, see Materials and Methods) for the DAB signal Control (normal) and Malignant (tumor) is shown.
(TIF)

S6 Fig ß-catenin (used at 1:100 dilution, Cy3-green) expression within the nucleus (stained blue with a DAPI counterstain) was measured in control normal and malignant (FOSSC) feline tissue. Tissue cores were imaged using a Zeiss Axioscan Z.1 slide scanner (Carl Zeiss) with a 40x magnification. Localisation of ß -catenin within the nucleus was measured on (A) benign and (B) tumor tissue cores, a random sample of epithelial tissue was selected and the total number of nuclei was counted along with the total number of nuclei which contain ß -catenin. A Box plot illustrates the percentage of ß -catenin in the nucleus for control (n = 5) and malignant (n = 11) individual tissue cores. The significance of difference between the benign and tumour samples was measured using a Mann-Whitney U test (* = *p*<0.01).
(TIF)

S1 Table True positive and true negative rates for putative FOSCC discriminatory markers from ROC curve analysis. Operating characteristics Sensitivity and 1-Specificity of the area fraction values for Cyclin D1, c-Myc and FRA1 at a specific value (Criteria >) calculated using ROC curves. AUC = area under the curve. An AUC of >0.7 is considered to provide adequate discrimination for a diagnostic marker. 95% CI intervals (sensitivity; 1-specificity) are: Cyclin D1 (0.72–0.91;0.73–0.89), c-Myc (0.66–0.89;0.63–0.82) and FRA1 (0.77–0.95;0.69–0.86). The data is tabulated from Fig 3. Youden index J for Cyclin D1, c-Myc and FRA1 was 0.66, 0.53 and 0.67, respectively.
(DOC)
