# Correction: Quantitative Expression and Co-Localization of Wnt Signalling Related Proteins in Feline Squamous Cell Carcinoma

**DOI:** 10.1371/journal.pone.0163838

**Published:** 2016-09-22

**Authors:** The PLOS ONE Staff

The affiliation for the eighth author is incorrect. Mariana Beltran is not affiliated with #7 but with #8 Queen’s Medical Research Institute, University of Edinburgh, Edinburgh, United Kingdom. The publisher apologizes for the error.
