# Supplementary figures and images for: Quantitative Expression and Co-Localization of Wnt Signalling Related Proteins in Feline Squamous Cell Carcinoma
Source: PLoS One. 2016 Aug 25;11(8):e0161103. doi: 10.1371/journal.pone.0161103 (PMC4999089; doi:10.1371/journal.pone.0161103)

Supplementary Figure S1 Fig

A

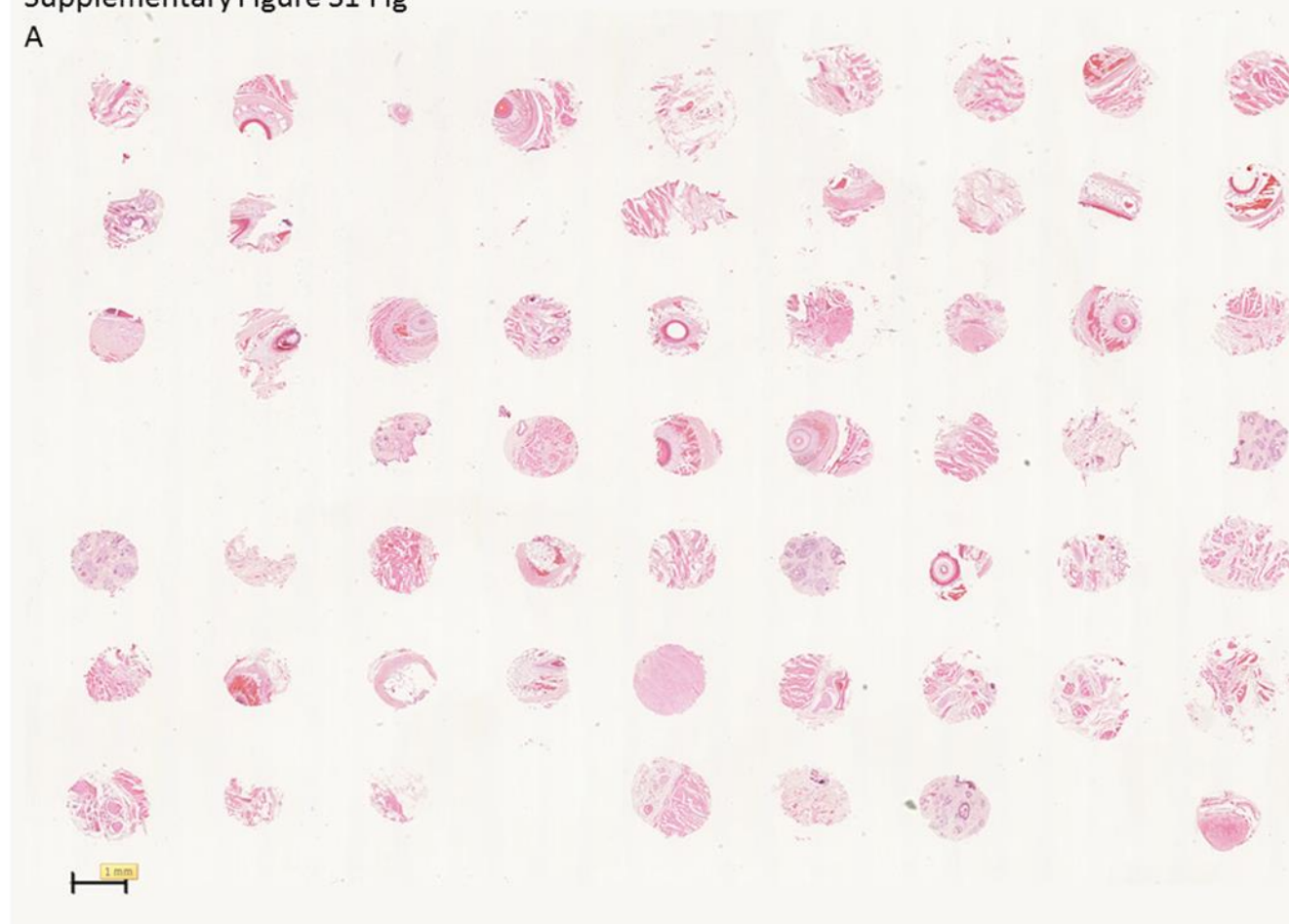

Supplementary Figure S1 Fig  
B

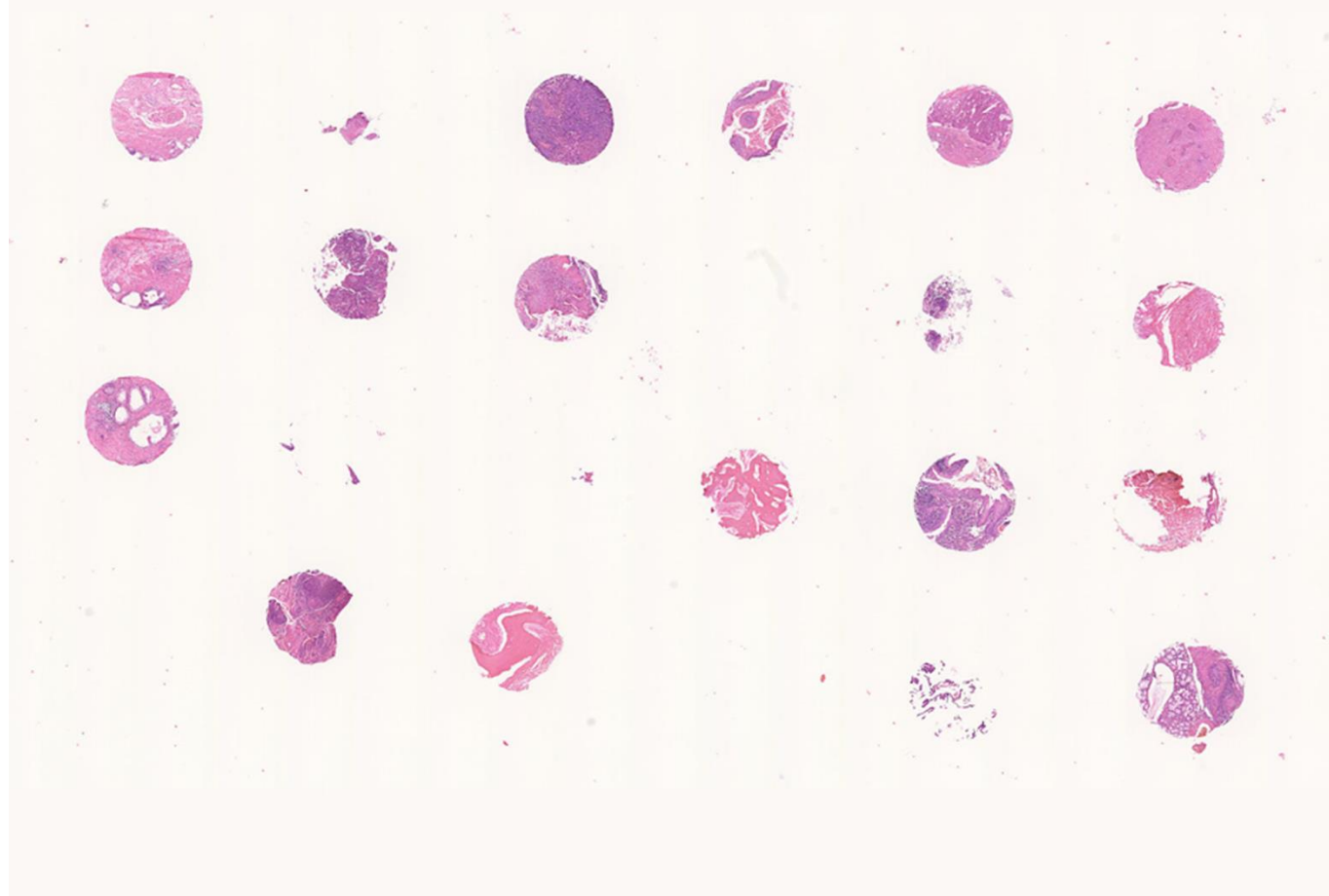

Supplementary Figure S1 Fig

C

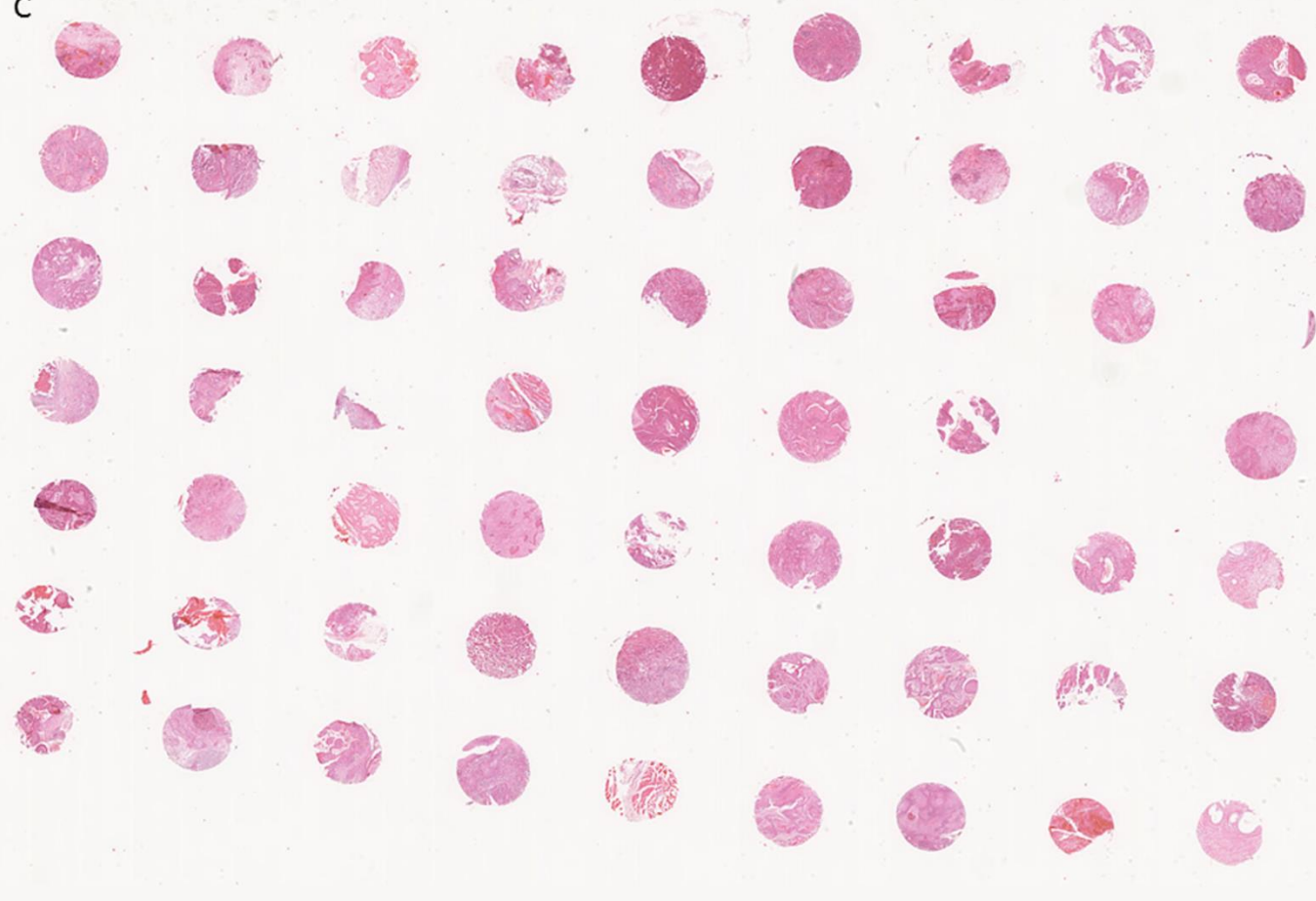

Supplement: S1 Fig — Blocks were arrayed with feline tissue samples and also with some human tissue samples for identification. The three slides used in this study (A, B and C) are stained with Haematoxylin & Eosin stain and scanned using a Hamamtsu Nanozoomer scanner are shown here. The original Nanozoomer (ndpi) files were viewed at higher resolution than shown in this figure histopathological analysis using the NDPI.view2 software. (PDF) [file pone.0161103.s001.pdf]

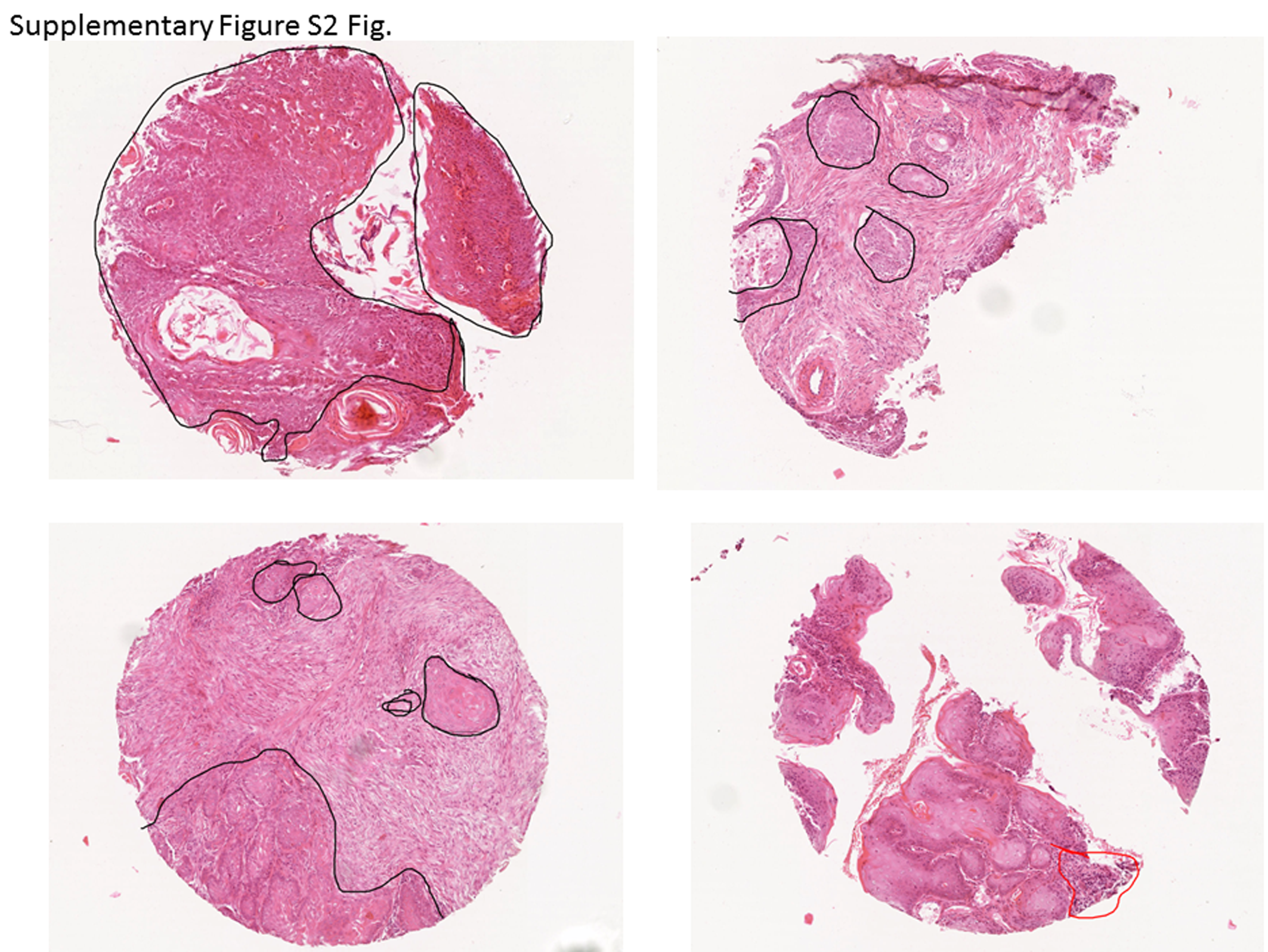

Supplement: S2 Fig — Tumor tissue is circled in black and inflammation in red. Present with a multifocal to coalescing pattern is a locally infiltrative, densely cellular neoplastic mass that infiltrates the surrounding stroma. Neoplastic cells are arranged in islands and sheets, within which they exhibit variable degrees of keratinization. Individual neoplastic cells are moderately large, polygonal with moderate to large amounts of cytoplasm. Nuclei are round to oval and contain prominent nucleoli. There is moderate degree of anisocytosis and anisokaryosis. The surrounding stroma exhibits variable amounts of fibrosis and inflammatory cell infiltrate. (TIF) [file pone.0161103.s002.tif]

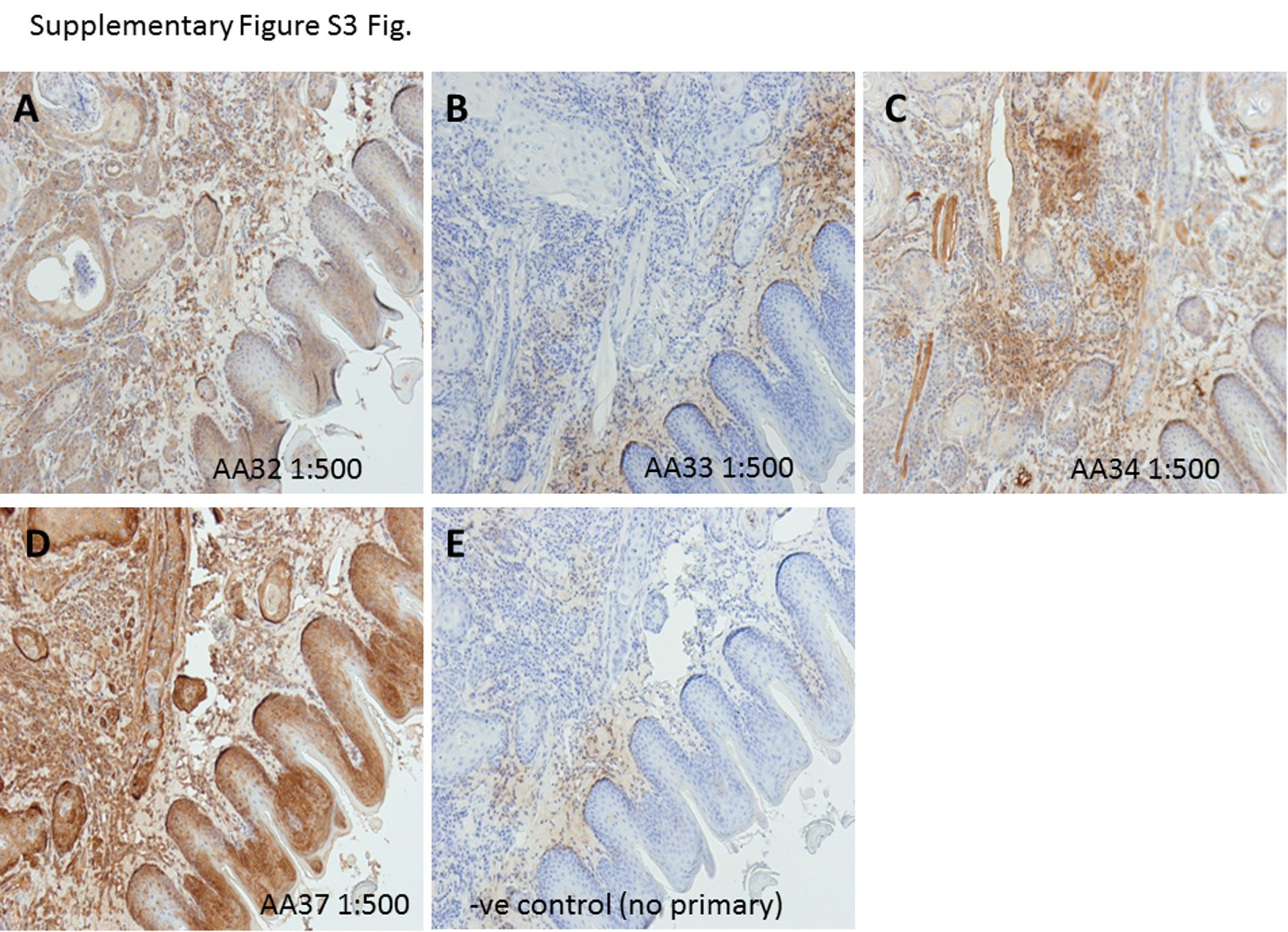

Supplement: S3 Fig — Images were acquired at 10x using a standard bright field microscope. The antibodies were coded (AA xx, A to D) and the experimenters blinded to their identity; negative controls without primary antibodies were used (E). Cross-reactivity of these antibodies was compared by using human tissues in which we have used these, previously (e.g. Arya et al, 2015). Antibody AA33 was not used as the DAB signal for this was similar to the no primary negative control (E). (TIF) [file pone.0161103.s003.tif]

Supplementary Figure S4 Fig.

(A)

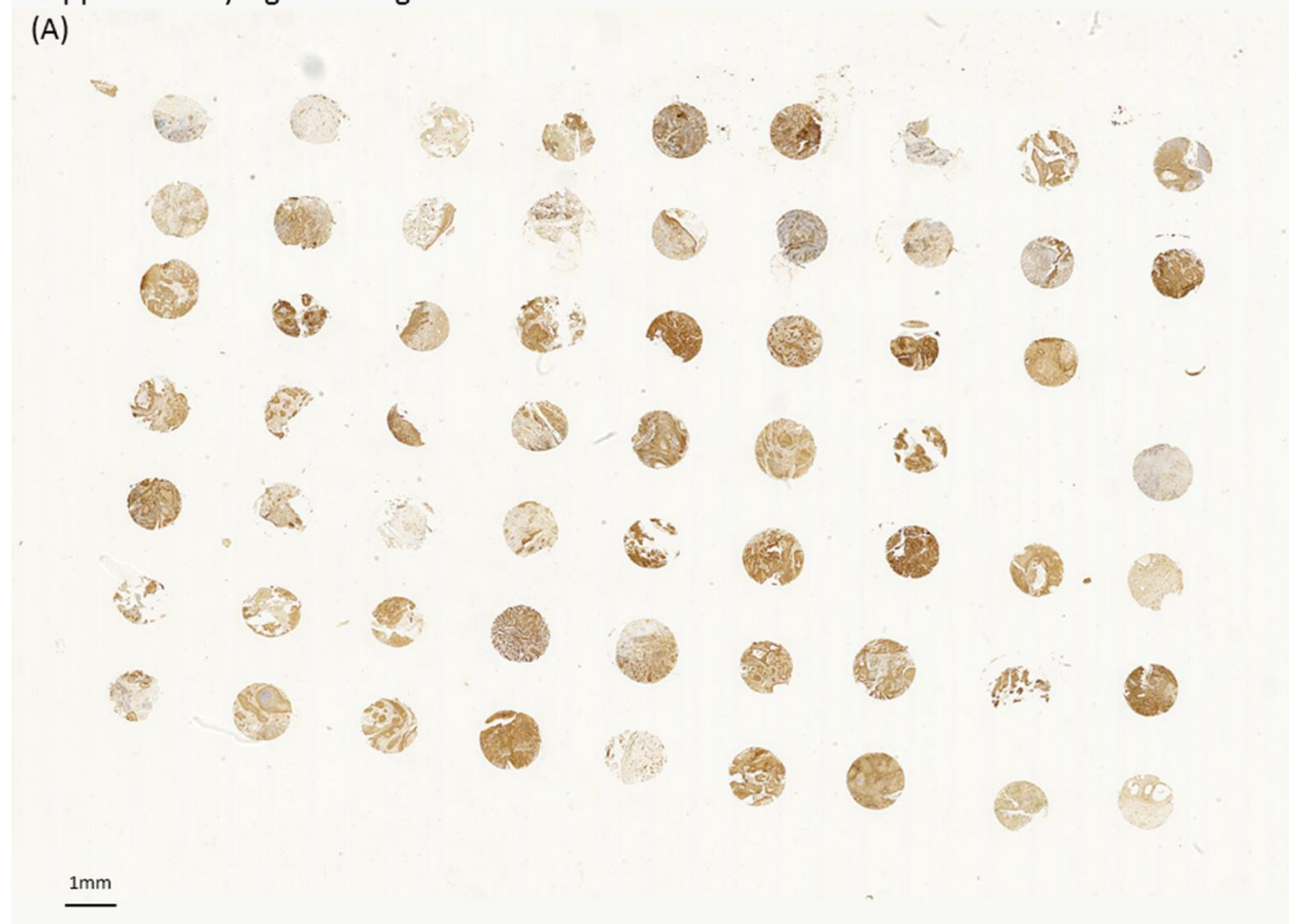

Supplementary Figure S4 Fig.  
(B)

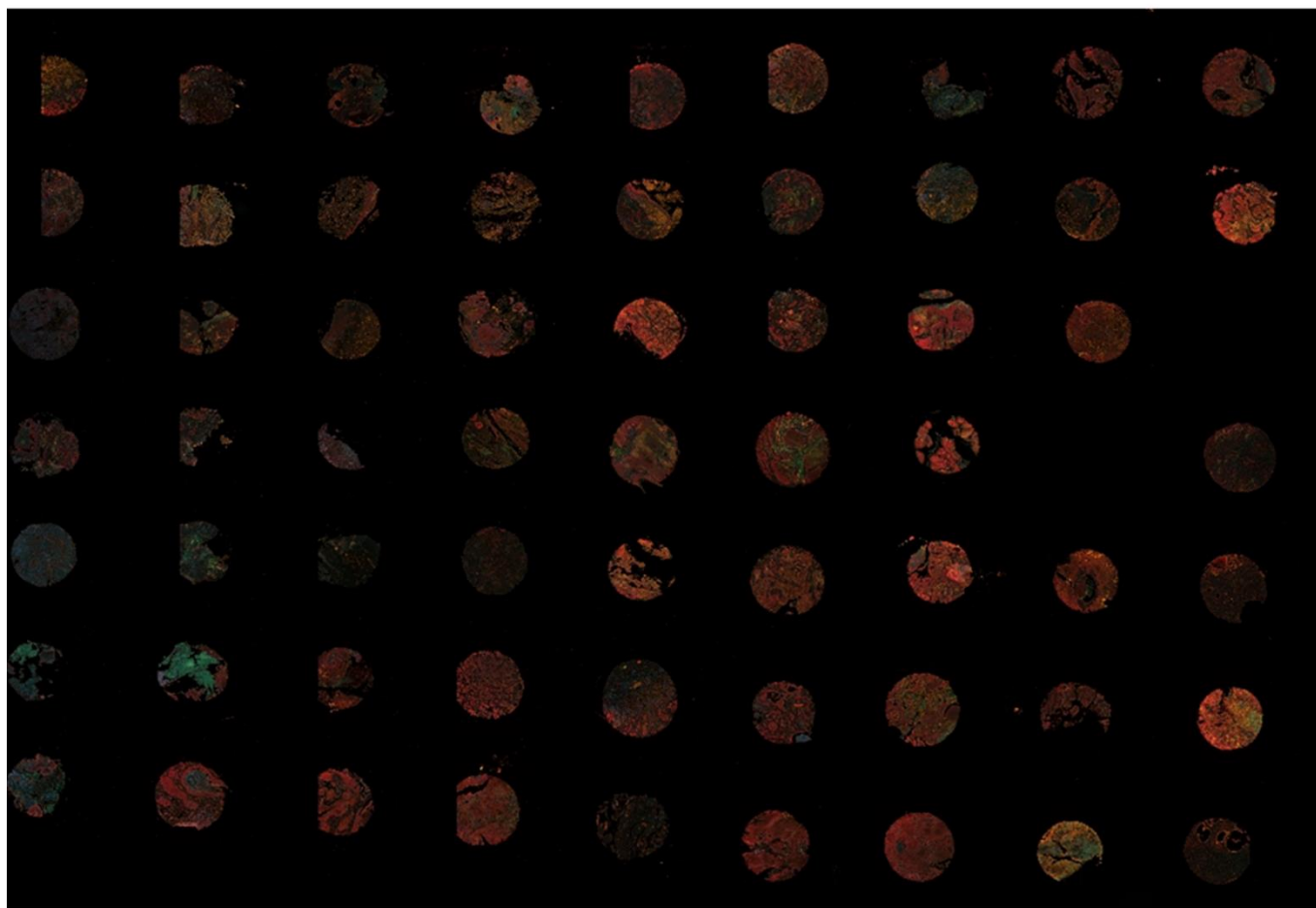

Supplement: S4 Fig — (A) The DAB stained slides were scanned using a Hamamtsu Nanozoomer scanner are shown here. The original Nanozoomer (ndpi) files were imaged using the NDPI.view2 software. at a higher resolution (1300 x 1300 pixels) than shown; these higher resolution images were used for quantitative analysis as described in Materials and Methods. (B) Multi-label tissue arrays were stained with three antibodies and counterstained with DAPI, simultaneously, and imaged using an Axioscan (Zeiss). The signal for the four fluorophores is shown in this composite image for one slide. (PDF) [file pone.0161103.s004.pdf]

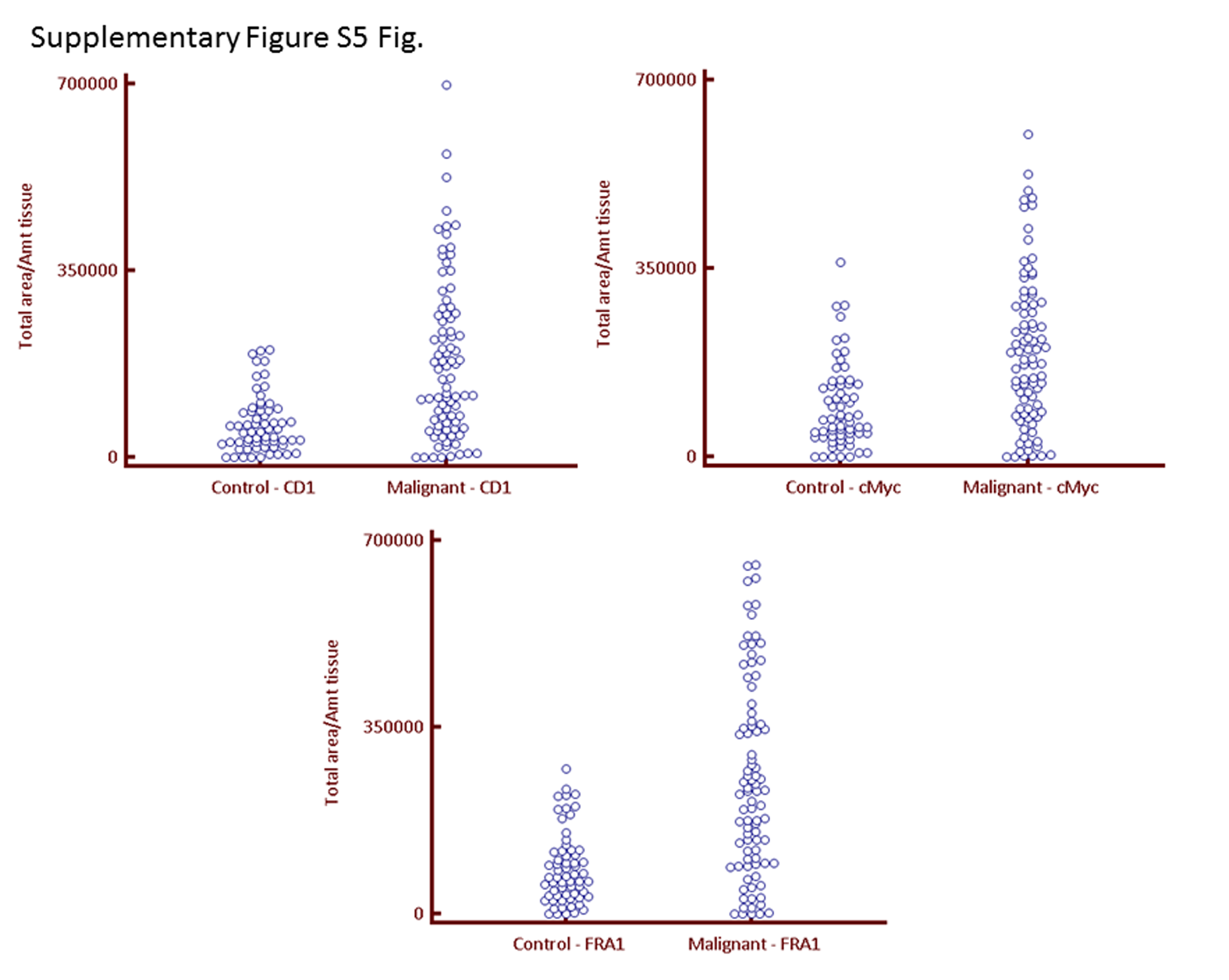

Supplement: S5 Fig — Total area (TA, see Materials and Methods) for the DAB signal Control (normal) and Malignant (tumor) is shown. (TIF) [file pone.0161103.s005.tif]

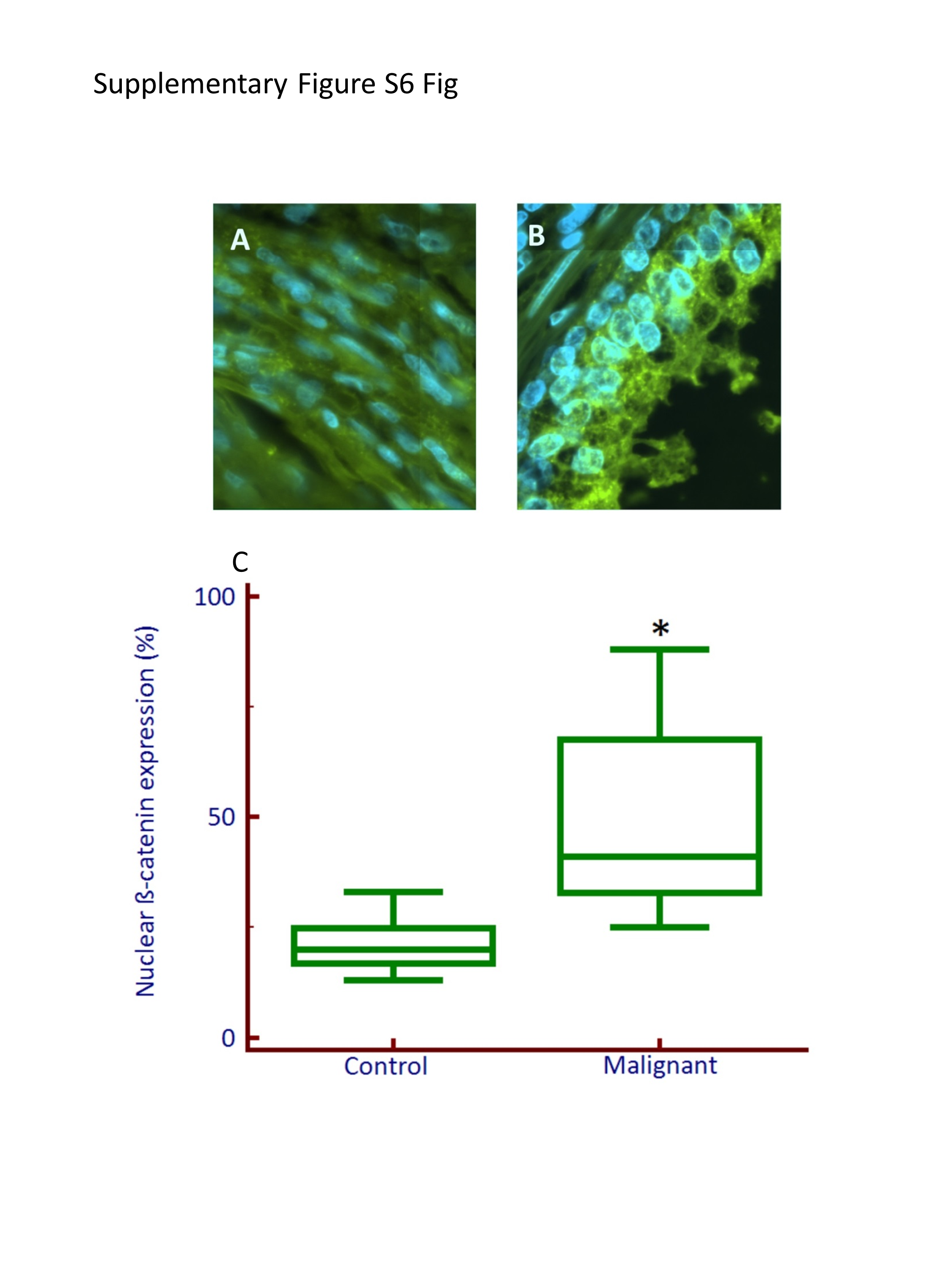

Supplement: S6 Fig — Tissue cores were imaged using a Zeiss Axioscan Z.1 slide scanner (Carl Zeiss) with a 40x magnification. Localisation of ß -catenin within the nucleus was measured on (A) benign and (B) tumor tissue cores, a random sample of epithelial tissue was selected and the total number of nuclei was counted along with the total number of nuclei which contain ß -catenin. A Box plot illustrates the percentage of ß -catenin in the nucleus for control (n = 5) and malignant (n = 11) individual tissue cores. The significance of difference between the benign and tumour samples was measured using a Mann-Whitney U test (* = p<0.01). (TIF) [file pone.0161103.s006.tif]
