# Supplementary material for: Quantitative Expression and Co-Localization of Wnt Signalling Related Proteins in Feline Squamous Cell Carcinoma
Source: PLoS One. 2016 Aug 25;11(8):e0161103. doi: 10.1371/journal.pone.0161103 (PMC4999089; doi:10.1371/journal.pone.0161103)
Supplement: S1 Table — Operating characteristics Sensitivity and 1-Specificity of the area fraction values for Cyclin D1, c-Myc and FRA1 at a specific value (Criteria >) calculated using ROC curves. AUC = area under the curve. An AUC of >0.7 is considered to provide adequate discrimination for a diagnostic marker. 95% CI intervals (sensitivity; 1-specificity) are: Cyclin D1 (0.72–0.91;0.73–0.89), c-Myc (0.66–0.89;0.63–0.82) and FRA1 (0.77–0.95;0.69–0.86). The data is tabulated from Fig 3. Youden index J for Cyclin D1, c-Myc and FRA1 was 0.66, 0.53 and 0.67, respectively. (DOC) [file pone.0161103.s007.doc]

Supplementary Table S1: **True positive and true negative rates for putative FOSCC discriminatory markers from ROC curve analysis**

|  | AUC | Criteria > | Sensitivity | 1-Specificity | + Likelihood ratios |
| --- | --- | --- | --- | --- | --- |
| Cyclin D1 | 0.88 | 3.4 | 0.84 | 0.80 | 4.5 |
| c-Myc | 0.81 | 3.5 | 0.79 | 0.73 | 2.9 |
| FRA1 | 0.89 | 3.5 | 0.88 | 0.78 | 4.2 |

Operating characteristics Sensitivity and 1-Specificity of the area fraction values for Cyclin D1, c-Myc and FRA1 at a specific value (Criteria >) calculated using ROC curves. AUC = area under the curve. An AUC of >0.7 is considered to provide adequate discrimination for a diagnostic marker. 95% CI intervals (sensitivity; 1-specificity) are: Cyclin D1 (0.72-0.91;0.73-0.89 ), c-Myc (0.66-0.89;0.63-0.82) and FRA1 (0.77-0.95;0.69-0.86). The data is tabulated from Figure 3. Youden index J for Cyclin D1, c-Myc and FRA1 was 0.66, 0.53 and 0.67, respectively.
